# Supplementary material for: A folate inhibitor exploits metabolic differences in Pseudomonas aeruginosa for narrow-spectrum targeting
Source: Nat Microbiol. 2024 Apr 9;9(5):1207–19. doi: 10.1038/s41564-024-01665-2 (PMC11087268; doi:10.1038/s41564-024-01665-2)
Supplement: Supplementary file 1 — Supplementary Fig. 1. [file 41564_2024_1665_MOESM1_ESM.pdf]

nature microbiology

Article

https://doi.org/10.1038/s41564-024-01665-2

A folate inhibitor exploits metabolic differences in *Pseudomonas aeruginosa* for narrow-spectrum targeting

In the format provided by the authors and unedited

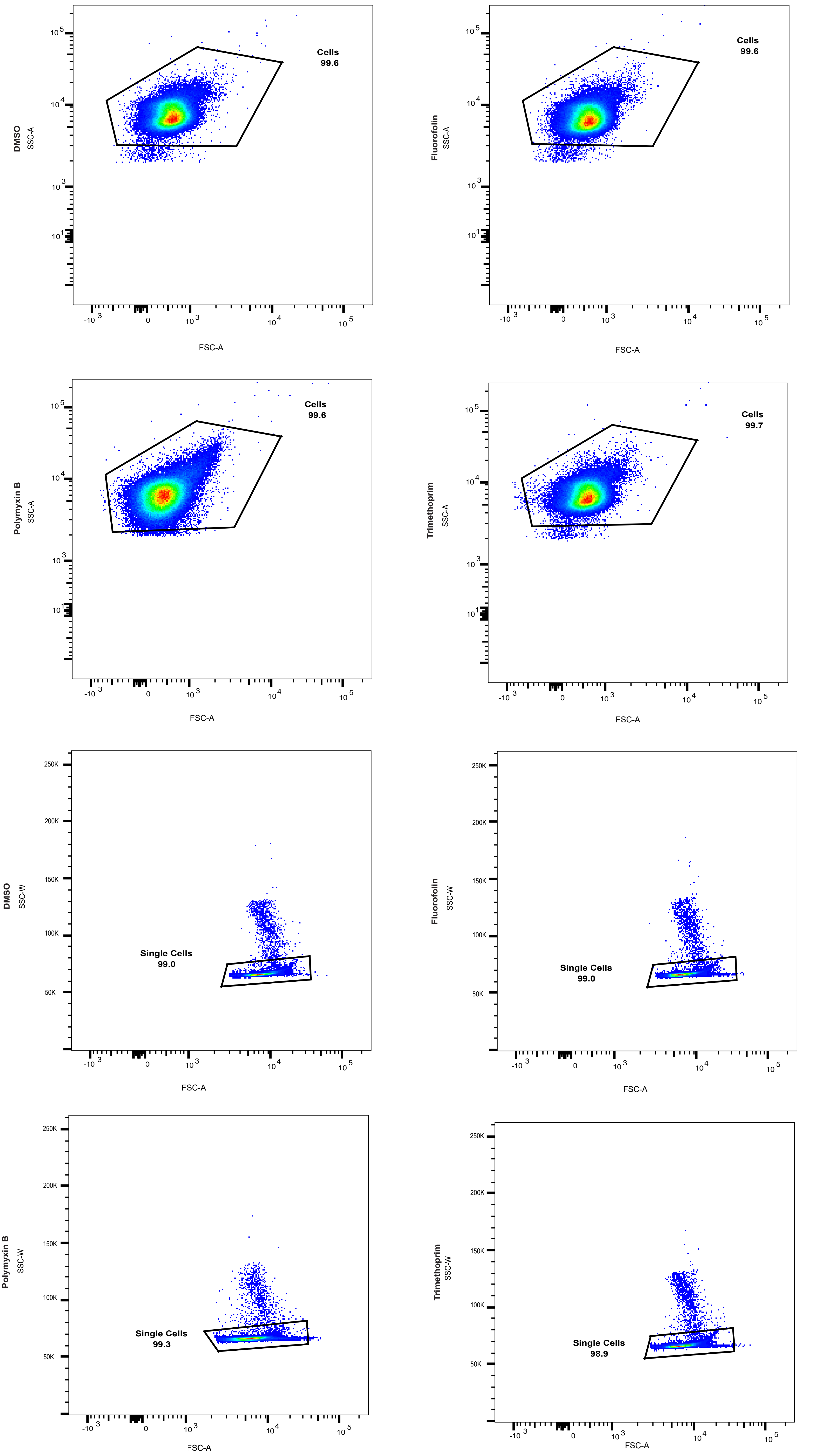

Supplementary Figure 1: Flow cytometry gating strategy. Cells were gated as live cells and singlets using FSC-A and SSC-W. From Single cells, gates were drawn for depolarization and permeabilization based on the posivite controls Polymixin B and CCCP as shown in Extended Data Figure 2. Trimethoprim and vehicle only (DMSO) controls were used as negative controls, and unstained controls (not shown) were included to confirm experimental setup.
