# Supplementary material for: A folate inhibitor exploits metabolic differences in Pseudomonas aeruginosa for narrow-spectrum targeting
Source: Nat Microbiol. 2024 Apr 9;9(5):1207–19. doi: 10.1038/s41564-024-01665-2 (PMC11087268; doi:10.1038/s41564-024-01665-2)
Supplement: Supplementary file 2 — Reporting Summary [file 41564_2024_1665_MOESM2_ESM.pdf]

nature portfolio

Corresponding author(s): Zemer Gitai

Last updated by author(s): Feb 7, 2024

Reporting Summary

Nature Portfolio wishes to improve the reproducibility of the work that we publish. This form provides structure for consistency and transparency in reporting. For further information on Nature Portfolio policies, see our Editorial Policies and the Editorial Policy Checklist.

Statistics

For all statistical analyses, confirm that the following items are present in the figure legend, table legend, main text, or Methods section.

n/a	Confirmed	
☐	☒	The exact sample size (*n*) for each experimental group/condition, given as a discrete number and unit of measurement
☐	☒	A statement on whether measurements were taken from distinct samples or whether the same sample was measured repeatedly
☐	☒	The statistical test(s) used AND whether they are one- or two-sided<br>*Only common tests should be described solely by name; describe more complex techniques in the Methods section.*
☐	☒	A description of all covariates tested
☐	☒	A description of any assumptions or corrections, such as tests of normality and adjustment for multiple comparisons
☐	☒	A full description of the statistical parameters including central tendency (e.g. means) or other basic estimates (e.g. regression coefficient) AND variation (e.g. standard deviation) or associated estimates of uncertainty (e.g. confidence intervals)
☐	☒	For null hypothesis testing, the test statistic (e.g. *F*, *t*, *r*) with confidence intervals, effect sizes, degrees of freedom and *P* value noted<br>*Give P values as exact values whenever suitable.*
☒	☐	For Bayesian analysis, information on the choice of priors and Markov chain Monte Carlo settings
☒	☐	For hierarchical and complex designs, identification of the appropriate level for tests and full reporting of outcomes
☒	☐	Estimates of effect sizes (e.g. Cohen's *d*, Pearson's *r*), indicating how they were calculated

Our web collection on statistics for biologists contains articles on many of the points above.

Software and code

Policy information about availability of computer code

Data collection	No software was used.
Data analysis	GraphPad Prism Version 9.4.1., FlowJo v10, and MATLAB R2022b were used for data analysis.

For manuscripts utilizing custom algorithms or software that are central to the research but not yet described in published literature, software must be made available to editors and reviewers. We strongly encourage code deposition in a community repository (e.g. GitHub). See the Nature Portfolio guidelines for submitting code & software for further information.

Data

Policy information about availability of data

All manuscripts must include a data availability statement. This statement should provide the following information, where applicable:

- Accession codes, unique identifiers, or web links for publicly available datasets
- A description of any restrictions on data availability
- For clinical datasets or third party data, please ensure that the statement adheres to our policy

The datasets generated during and/or analyzed during the current study are available from the corresponding author on reasonable request. Links to raw data for metabolomics and RNA sequencing depositories are provided in the final submission of the manuscript (GEO (GSE249862) and MassIVE (MSV000093598)).

Human research participants

Policy information about studies involving human research participants and Sex and Gender in Research.

Reporting on sex and gender	N/A
Population characteristics	N/A
Recruitment	N/A
Ethics oversight	N/A

Note that full information on the approval of the study protocol must also be provided in the manuscript.

Field-specific reporting

Please select the one below that is the best fit for your research. If you are not sure, read the appropriate sections before making your selection.

☒ Life sciences ☐ Behavioural & social sciences ☐ Ecological, evolutionary & environmental sciences

For a reference copy of the document with all sections, see nature.com/documents/nr-reporting-summary-flat.pdf

Life sciences study design

All studies must disclose on these points even when the disclosure is negative.

Sample size	Sample size calculations were not performed. Number of replicates in reported in relevant figures. For C. elegans experiments, n were chosen based on similiar stuides in the literature (Kaletsky et al 2020). Sample size for mouse experiments was chosen based on previous mouse infection model experiments performed at University of North Texas Health Science Center. For murine pharmacokinetic experiments, n=3 was used for determination of drug properties. For murine infection experiments, n=5 per group was used.
Data exclusions	C. elegans data was excluded from this study if a worm could not be located on a given plate (this worm would be censored from any subsequent analysis). Only worms that were confirmed dead or alive were included in the analysis. All other data was included in this study.
Replication	Experiments were repeated in biological duplicate or triplicate and, at minimum, in technical duplicate to confirm reproducibility. All attempts at replication were successful. Animal experiments were performed using multiple mice in randomly allocated groups.
Randomization	Organisms were allocated to each group randomly.
Blinding	Blinding was not relevant to this study as all outcomes were objective measurements and therefore were not biased by labeled groups.

Reporting for specific materials, systems and methods

We require information from authors about some types of materials, experimental systems and methods used in many studies. Here, indicate whether each material, system or method listed is relevant to your study. If you are not sure if a list item applies to your research, read the appropriate section before selecting a response.

Materials & experimental systems

n/a	Involved in the study	
☒	☐	Antibodies
☐	☒	Eukaryotic cell lines
☒	☐	Palaeontology and archaeology
☐	☒	Animals and other organisms
☒	☐	Clinical data
☒	☐	Dual use research of concern

Methods

n/a	Involved in the study	
☒	☐	ChIP-seq
☐	☒	Flow cytometry
☒	☐	MRI-based neuroimaging

Eukaryotic cell lines

Policy information about cell lines and Sex and Gender in Research

Cell line source(s)	HLF: human lung fibroblast (Cell Applications 506K-05a), HK-2: human kidney epithelial (ATCC-CRL2190), PBMC: peripheral

Cell line source(s)	blood mononuclear cell (TPCSP-B010C), WI-38: Embryonic lung tissue (ATC-CCL-75) provided by Pharmaron, Inc. (Beijing, ROC).
Authentication	Authentication of cell lines was performed by Pharmaron, Inc. (Beijing, ROC) using STR profiling.
Mycoplasma contamination	Mycoplasma testing was performed by Pharmaron, Inc. (Beijing, ROC). All cell lines tested were negative for mycoplasma.
Commonly misidentified lines (See ICLAC register)	None

Animals and other research organisms

Policy information about studies involving animals; ARRIVE guidelines recommended for reporting animal research, and Sex and Gender in Research

Laboratory animals	Male CD-1 mice aged 6–8 weeks old and female CD-1 mice aged 5–6-weeks old were used in this study. All procedures were conducted in accordance with a protocol approved by the UNTHSC Animal Care and Use Committee (IACUC). Animals were housed with rooms undergoing ten to fifteen air changes per hour. Air provided to the animal rooms was controlled for temperature and humidity and is fully monitored, 24 hours a day. Air pressures was balanced according to room use. Lighting was controlled in individual rooms by automatic timers with a standard 12 hour on – 12 hour off cycle.
Wild animals	The study did not involve wild animals.
Reporting on sex	Male CD-1 mice were used for PK studies. Female CD-1 mice were used for MTD and thigh infection model. The mice are housed individually with free access to food and water during the study. Animals will be cared for in accordance with Guide for Care and Use of Laboratory Animals" (National Academy Press, Washington DC, 2011).
Field-collected samples	This study did not involved samples collected from the field.
Ethics oversight	Pharmaron Inc. carried out PK studies according to industry standards (Beijing, ROC). University of North Texas Health Science Center (Fort Worth, Texas) carried out infection model and MTD studies under UNTHSC approved (IACUC) protocols# IACUC-2021-0003 and IACUC-2020-0039. All procedures were conducted in accordance with a protocol approved by the UNTHSC Animal Care and Use Committee (IACUC)

Note that full information on the approval of the study protocol must also be provided in the manuscript.

Flow Cytometry

Plots

Confirm that:

☒ The axis labels state the marker and fluorochrome used (e.g. CD4-FITC).

☒ The axis scales are clearly visible. Include numbers along axes only for bottom left plot of group (a 'group' is an analysis of identical markers).

☒ All plots are contour plots with outliers or pseudocolor plots.

☒ A numerical value for number of cells or percentage (with statistics) is provided.

Methodology

Sample preparation	Overnight P. aeruginosa PA14 or E. coli lptD4213 cultures were diluted 1:100 and grown to mid exponential phase at 37°C. Cultures were diluted 1:10 into PBS and treated with antibiotics for 15 minutes. P. aeruginosa PA14 was stained with TO-PRO-3 (640 nm excitation, 670/30 nm emission) to measure cell membrane integrity. E. coli lptD4213 was stained with both TO-PRO-3 and DiOC2(3)
Instrument	LSRII flow cytometer BD Biosciences
Software	FlowJo v10 software (FlowJo LLC, Ashland, OR)
Cell population abundance	For each assay 100,000 events were collected and cell population % is reported in each figure.
Gating strategy	Gates for depolarization were determined using CCCP as a positive control. Gates for permeabilization were determined using Polymixin B (Sigma-Aldrich P1004) and untreated controls.

☒ Tick this box to confirm that a figure exemplifying the gating strategy is provided in the Supplementary Information.
